# Regulating Androgen Receptor Function in Prostate Cancer: Exploring the Diversity of Post-Translational Modifications

**DOI:** 10.3390/cells13020191

**Published:** 2024-01-19

**Authors:** Lance Edward V. Lumahan, Mazia Arif, Amy E. Whitener, Ping Yi

**Affiliations:** 1 Department of Molecular and Cellular Biology, Baylor College of Medicine, Houston, TX 77204, USA; 2 Center for Nuclear Receptors and Cell Signaling, Department of Biology and Biochemistry, University of Houston, Houston, TX 77205, USA

**Keywords:** androgen receptor, post-translational modifications, prostate cancer, acetylation, methylation, phosphorylation, ubiquitination, sumoylation

## Abstract

Androgen receptor (AR) transcriptional activity significantly influences prostate cancer (PCa) progression. In addition to ligand stimulation, AR transcriptional activity is also influenced by a variety of post-translational modifications (PTMs). A number of oncogenes and tumor suppressors have been observed leveraging PTMs to influence AR activity. Subjectively targeting these post-translational modifiers based on their impact on PCa cell proliferation is a rapidly developing area of research. This review elucidates the modifiers, contextualizes the effects of these PTMs on AR activity, and connects these cellular interactions to the progression of PCa.

## 1. Introduction

**Figure 1:**
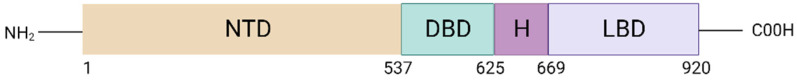
Domain organization of AR. Created with BioRender.

Prostate cancer (PCa) is the second leading cause of cancer mortality among men in the United States [1]. High levels of AR expression are associated with aggressive PCa [2,3,4]. Suppressing androgen receptor activity through different forms of androgen deprivation therapy (ADT) has been proven to be an effective clinical strategy in targeting PCa. This clinical strategy has been adopted as the standard of care for treating PCa patients [5]. However, many patients treated with this therapy develop castration resistance (CRPC) [6]. Aside from non-AR dependent mechanisms such as expression of the glucocorticoid receptor [7], AR reactivation remains as an important mechanism for CRPC. Elucidating how oncogenic AR functions without androgen is a rapidly developing research focus [8]. The structural domains of AR have been well-defined. AR consists of an N-terminal regulatory domain (NTD), a DNA binding domain (DBD), a hinge region (H), and a ligand-binding domain (LBD) (Figure 1) [9]. The NTD contains a ligand-independent activation function (AF1) domain. This domain contributes the most to AR function [10] and is responsible for recruiting coactivators SRC-3/NCOA3 (steroid receptor coactivator-3) and p300 [11]. The LBD contains a ligand-dependent activation function (AF2), which interacts with coactivators via LXXLL (leucine-X-X-leucine-leucine) motifs [10]. Several clinically relevant splice variants (such as AR-V7) that lack an LBD have been found in prostate tumor cells [12]. The presence of this splice variant in patient-circulating tumor cells has been associated with enzalutamide and abiraterone resistance [12].

Upon androgen binding, cytosolic AR undergoes a conformational change that releases LBD-bound heat-shock proteins (HSPs) including HSP90 [13]. AR homodimerizes and translocates into the nucleus [14], where the AR binds to androgen response elements (AREs), conserves DNA sequences within promoter/enhancer regions of AR target genes [10], and subjectively recruits coregulators (coactivators or corepressors) to regulate target gene expressions [15]. AR-V7 can reside constitutively in the nucleus, and this protects the receptor from enzalutamide’s ability to attenuate AR nuclear localization and prevent cell proliferation [12].

Post-translational modifications (PTMs) strongly influence the ability of AR to activate transcription. Subjectively influencing these PTMs is a rapidly developing research area aimed towards supplementing current anti-androgen therapies in PCa. AR can be acetylated, methylated, phosphorylated, ubiquitinated, and/or SUMOylated. Here, we comprehensively review how each PTM impacts the transcriptional activity of AR and contributes to prostate carcinogenesis.

## 2. Acetylation

Protein acetylation is one of the major PTMs regulating protein functions. This PTM is catalyzed by histone acetyltransferases (HATs) and involves the attachment of the acetyl group from acetyl-CoA to the ε-amino group of lysine, which neutralizes the positive charge of lysine. There are three acetylation sites located in the hinge region of AR, including K631, K633, and K634 (formerly K630, K632, and K633); these create a motif that is acetylated by HAT p300 and the p300/cAMP-response element-binding protein-associated factor (PCAF) [16]. This acetylation level was reported to increase upon ligand dihydrotestosterone (DHT) stimulation or histone deacetylase (HDAC) inhibitor trichistatin (TSA) treatment in PCa cell lines. AR acetylation increases cell proliferation and decreases cell death [17]. When ligand-bound AR translocates into the nucleus, it binds to ARE and recruits coregulators such as HATs. The recruited HATs then acetylate AR [16]. Both in vivo (in cells and mice) and in vitro studies have demonstrated the p300- and PCAF-mediated acetylation of AR [17,18,19,20].

p300- and PCAF-mediated AR acetylation regulates both basal and ligand-induced AR function [17]. p300 and PCAF are AR coactivators. p300 directly interacts with AR and recruits PCAF to the AR complex [11,19,20]. Using an in vitro ^14^C-acetylation assay and mass spectrometry, Fu et al. demonstrated that both p300 and PCAF acetylate AR (specifically, PCAF acetylates K633 and K634), while p300 appears mainly to acetylate K631 [17,18]. Overexpression of PCAF increases DHT-induced AR activity, while K633/634 mutation greatly reduces DHT-induced activation. That study also showed that PCAF HAT activity is required for full induction of AR activity, and that deletion of the PCAF HAT domain reduced AR-induced activity by 45% [18]. AR K631A mutation also similarly abolished DHT-induced AR activity [18]. Mechanistically, AR acetylation enhances its association with p300 and reduces the binding to corepressors HDAC1 (histone deacetylase 1) and NCoR (nuclear receptor corepressor) [17]. All these pieces of evidence suggest that in each of these AR lysine residues, acetylation is important for full induction of AR activity by DHT. Consistent with its effect on AR transcriptional activity, AR acetylation was also shown to promote PCa cell proliferation and contact-independent growth, both in the presence and absence of DHT [17]. In the absence of the ligand, AR acetylation plays a role in regulating cell-cycle genes, decreasing cell death through MEKK1-dependent apoptosis in response to TRAIL and cycloheximide [17].

The three lysine residues mentioned above are also acetylated by HIV-Tat interactive protein 60 kDa (Tip60), as demonstrated by both in vitro and in vivo (in cells and mice) studies [21,22,23,24]. Tip60 is a MYST histone acetyltransferase, which is involved in many processes including chromatin remodeling, cell cycle progression, nuclear receptor signaling, and DNA damage repair [25]. It is a coactivator and interacts with AR through the LXXLL motif, a nuclear receptor interaction motif found in many nuclear receptor coregulators [22]. Similar to p300 and PCAF, Tip60 acetylates AR directly at K631/633/634 [23]. The wild-type—but not the HAT defective mutant—of Tip60 increased AR-mediated transcription [23]. Mutations in either K631 or K633/634 abolished Tip60-promoted AR transcriptional activity in response to androgen, suggesting that Tip60 may acetylate all three lysine residues [23]. Acetylation of AR at these lysine residues also regulates AR intracellular localization. The acetylation-mimicking mutants were found mainly in the nucleus despite androgen starvation, while the non-acetylation-mimicking mutants were in the cytoplasm despite androgen stimulation [24]. Tip60 knockdown decreased the proliferation of different AR-positive PCa cell lines (including CRPC cells) but not AR-negative PC-3 cells [24]. Tip60 overexpression in CRPC cells resulted in increases in the acetylated form of AR as well as AR localization in the nucleus even without androgen [24]. Other studies also showed that Tip60 is mainly found in the nucleus of hormone refractory PCa. When androgens were depleted, an upregulation of Tip60 was observed both in vitro and in vivo [21]. These studies indicate the role of Tip60 in the progression of PCa to CRPC.

Besides the three lysine residues in the hinge region of AR, acetylation also occurs in the DNA binding domain at K618, which has been demonstrated in mouse, cell, and in vitro experiments [26,27]. This residue acetylation is mediated by Arrest-defect-1 protein (ARD1). ARD1 is an N-acetyltransferase that plays a role in many cellular processes including apoptosis, cell proliferation, and cell-cycle arrest [28]. ARD1 along with Hsp90 form a complex with AR, which then acetylates AR at K618 [25,26,28]. Studies using acetylation-defective K618R and acetylation-mimicking K618Q mutants demonstrated that K618 acetylation promotes RNA Pol II binding, AR transcriptional activity, PCa cell growth, and xenograft tumor formation [26]. Mechanistically, when androgen is present, the levels of ARD1 and ARD1-mediated AR acetylation increase. This results in the dissociation of AR from HSP90, leading to translocation of acetylated AR into the nucleus for gene activation [26,28]. ARD1 is upregulated by androgen in an AR-dependent manner in PCa and found to be critical for transcriptionally activating AR target genes that are involved in prostate tumorigenesis, both in vivo and in vitro [27].

In contrast to the functions of HATs, the histone deacetylase SIRT1 has been shown to deacetylate AR and reduce AR function. Both cell and in vitro studies show that SIRT1 interacts with AR and represses the function of p300 and PCAF by deacetylating AR [29]. LNCaP cells transduced with MSCV-SIRT1 formed fewer and smaller colonies in soft agar when compared to controls, suggesting that SIRT1 inhibits cell proliferation and contact-independent growth [29]. Through deacetylation, SIRT1 represses AR-mediated transcription and is needed for (AR antagonist) bicalutamide-mediated repression of PCa cell proliferation [30].

## 3. Methylation

The lysine-rich sequence (KLKK) located in the hinge domain of AR (which is the target site for the p300-, PCAF-, and Tip60-mediated acetylation mentioned above) is also a conserved target site found in several proteins for SET9-mediated methylation [31]. SET9 is a histone methyltransferase known to methylate histone H3 K4 residue, and it potentiates transcription activation. The resemblance between the lysine-rich sequence targeted by SET9 and the KLKK motif of the AR hinge domain indicates a potential role of methylation in AR regulation. The lysine residues K631 and K633 within the AR KLKK motif are directly modified by SET9. This modification enhances N- and C-termini inter-domain interactions that are crucial for AR transcriptional activity and target gene expression [31,32]. In addition, SET9 nuclear expression is found to be notably upregulated in the malignant epithelium compared to benign prostate tissue, suggesting that SET9 is a positive regulator of AR function, and that deregulation of SET9 expression may drive uncontrolled cell proliferation in PCa [31].

The *TMPRSS2:ERG* gene fusion is one of the seven major molecular subtypes of AR-positive PCa [33,34]. The transcription factor ERG has been shown to recruit PRMT5 (protein arginine methyltransferase 5) to AR-target genes where it methylates AR on arginine 761. R761 methylation attenuates ligand-dependent AR activation, most likely by altering interactions between the DBD and LBD. This represses AR recruitment and downregulates the transcription of AR target genes involved in lineage differentiation in prostate epithelium. Mutation of R761 leads to a transcriptionally hyperactive AR. It is believed that the proliferative effects of ERG and PRMT5 are mediated at least in part through coaxing AR towards genes that would induce proliferation rather than differentiation [35].

AR acetylation and methylation also regulate lncRNA recruitment to AR. LncRNA *PRNCR1* and *PCGEM1* are highly expressed in aggressive PCa. *PRNCR1* and *PCGEM1* interact with AR in a K631/634 acetylation- and K349 methylation-dependent manner, respectively. The binding of *PRNCR1* to the C-terminal acetylated AR (K631/K634) recruits the methyltransferase DOT1L; this methylates K349 residue, which is critical for the recruitment of the second lncRNA, *PCGEM1*, to the AR N-terminus. These lncRNA recruitments do not affect AR binding to target gene enhancers but they are important for the cohesin-dependent formation of chromatin loops between enhancer and promoter. ChIP-3C experiments demonstrated that depletion of either *PRNCR1* or *PCGEM1* impaired the enhancer: promoter interaction of AR target genes. In CRPC cells, these overexpressed lncRNAs can interact with both C-terminal truncated (AR-V7) and full-length AR, causing ligand-independent activation of the AR-regulated transcriptional program and tumor cell proliferation. shRNA-mediated knockdown of these lncRNAs in CRPC cell lines significantly suppressed tumor xenograft growth in vivo [36].

## 4. Phosphorylation

**Figure 2:**
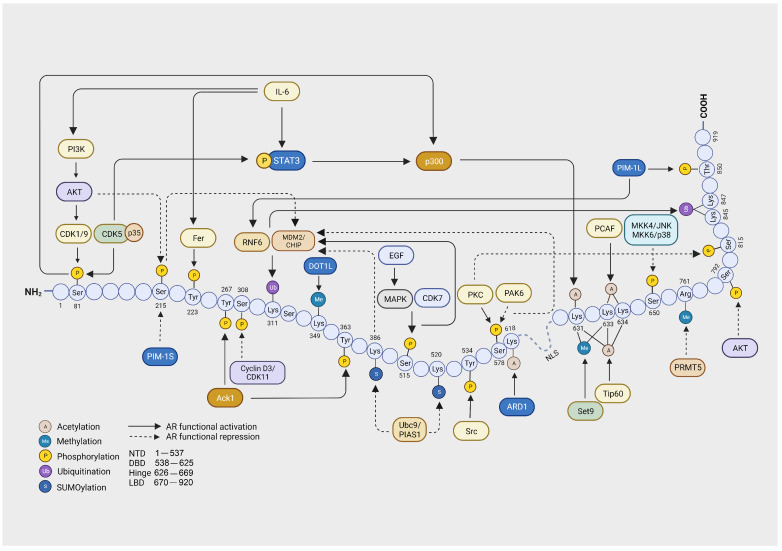
Diagrammatic representation of post-translational modifications of androgen receptor (AR), including acetylation, methylation, phosphorylation, ubiquitination, and SUMOylation. Solid arrows represent modifications that functionally activate AR activity, whereas dotted arrows represent modifications that functionally repress AR activity. N-terminal domain (NTD), DNA binding domain (DBD), and ligand binding domain (LBD). Created with BioRender.

Phosphorylation accounts for most of the post-translational modifications of AR, and at least 19 phosphorylation sites have been identified so far. Most of these residues are phosphorylated in the presence of androgen (testosterone or dihydrotestosterone), whereas some of the phosphorylation sites are androgen-independent [37,38]. Most of this phosphorylation has been found to occur in serine, threonine, and tyrosine residues and regulates AR transactivation, cellular localization, and protein stability [39,40,41]. Below, we group the phosphorylation sites according to their functions, discuss the kinases that have been recognized for these sites, and provide insights into the function and regulation of phosphorylation (Figure 2).

### 4.1. Positive Regulation of AR Transcriptional Activity

Serine 81 is the most extensively studied phosphorylation site in the NTD of AR, and phosphorylation at this site has been found to promote AR transcriptional activity [39,42,43,44]. Recent studies using an AR-phosphorylated S81-specific antibody revealed that S81 phosphorylation is associated with androgen-dependent AR transcriptional activation and this site is hypo-phosphorylated in DNA-binding-defective AR mutants [43]. A considerable number of studies have primarily focused on kinases that phosphorylate AR leading to AR transactivation. So far, many different kinases have been reported to play a role in S81 phosphorylation. Among them, members of the cyclin-dependent kinase (CDK) family have been widely investigated, including CDK1, CDK2, CDK5, and CDK9 [39,42,43,44,45,46,47]. Activation of CDK1 via cyclin B results in a notable upregulation in AR S81 phosphorylation, whereas the use of CDK1 inhibitors diminishes both AR S81 phosphorylation and transcriptional activity in PCa cells. This observation aligns with the significantly increased expression of cyclin B and CDK1 in androgen-independent PCa cells. In androgen-independent C4-2 PCa cells, roscovitine (a known CDK inhibitor) treatment revoked responses to low androgen levels, emphasizing that increased CDK1 levels could sensitize CRPC cells to minimal androgen levels [39].

Further investigations into the role of CDK9-mediated AR phosphorylation revealed that while CDK1 induces a modest level of S81 phosphorylation, CDK9 emerges as the predominant kinase responsible for S81 phosphorylation in response to androgen [44,45,48]. The primary function of CDK9/cyclin T complex (also referred to as positive transcriptional elongation factor b, P-TEFb) is to phosphorylate RNA polymerase II and promote transcription elongation. CDK9/cyclin T remains in an inactive state when complexed with 7S RNA and HEXIM1. Dephosphorylation of CDK9 by serine/threonine phosphatase PP1α dissociates CDK9 from the inhibitory complex and activates the kinase [49]. It was reported that AR recruits PP1α through the LBD to the chromatin. The recruited PP1α activates CDK9, leading to AR S81 phosphorylation, p300 recruitment, histone acetylation, BRD4 (bromodomain-containing-protein 4) binding, and the subsequent recruitment of P-TEFb, generating a positive feedback loop that sustains transcription [48].

Recently, a reciprocal crosstalk between AR signaling and PI3K pathway has been highlighted as a potential mechanism underlying CRPC [50]. Alterations in PI3K signaling in advanced PCa are predominantly driven by a loss of the tumor suppressor gene PTEN which accelerates progression in approximately 70% of metastatic PCa [51]. The absence of PTEN or the activation of PI3K leads to AKT phosphorylation and activation [52,53,54]. Inhibition of the PI3K-AKT pathway activates AR by attenuating the negative feedback inhibition of HER kinases, while AR inhibition promotes the PI3K activity via reducing levels of AKT phosphatase PHLPP in PTEN-less PCa [50]. In a mouse model of PTEN-deletion-induced PCa, AR protein is substantially down-regulated compared to wild-type. Genetic ablation of p300, which acts as a coactivator for many transcription factors including AR, p53, and NFκB, further attenuated the levels of AR in the PTEN-deficient prostates. This finding (that p300 is required for AR protein maintenance) in PTEN-deficient mouse prostate tumors was also confirmed in human PCa cells in which PTEN expression was abolished by RNA interference. A positive correlation between expression levels of p300 and AR was also found in human PCa specimens. Mechanistically, it was reported that PTEN loss activates CDKs, which in turn increases AR phosphorylation at S81. S81 phosphorylation then promotes p300 recruitment, resulting in AR acetylation. It was further demonstrated that p300-mediated AR acetylation inhibits AKT-activated AR polyubiquitination, thereby preventing its protein degradation [55].

Altogether, these studies indicate that CDK1 and CDK9-mediated AR S81 phosphorylation promotes p300 recruitment, AR acetylation, and protein stabilization. Recruited p300 further promotes histone acetylation and BRD4 binding, thereby enhancing AR transcriptional activity. In contrast, blocking S81 phosphorylation significantly suppresses AR transcriptional activity [48].

Tyrosine 223 is another phosphorylation site located in the AR TAU1 region (transactivation unit 1, a region between residues 101 and 370 in the NTD that is indispensable for full AR activity [56] and known to play a pivotal role in AR transactivation). Fer tyrosine kinase is reported to mediate IL-6-induced androgen-independent AR transactivation by phosphorylating Y223 via its SH2 domain. IL-6 stimulated AR activation is one important mechanism driving androgen-independent PCa cell growth. Fer-mediated AR Y223 phosphorylation was found to play an important role in IL-6-promoted AR target gene expression and growth response in PCa cells, and it contributes to aberrant AR signaling via crosstalk with pSTAT3 during CRPC progression [57,58].

On the other hand, serine 515 phosphorylation in the NTD represents a unique regulation mechanism. This site is phosphorylated by the transcription factor TFIIH, via its subunit CDK7 kinase. Impaired phosphorylation at S515 or CDK7 silencing disrupts AR transactivation. It was found that S515 phosphorylation facilitates the recruitment of an E3 ubiquitin ligase MDM2 (mouse homolog of double minute 2), which promotes AR polyubiquitination and proteasomal degradation [59]. Contrary to the traditional view of protein degradation effects on protein activity, MDM2-mediated AR degradation promotes the cyclic recruitment of AR to the target gene promoter, and it is a key step for an accurate AR transactivation. Non-phosphorylated AR S515A mutant recruits a different E3 ubiquitin ligase CHIP (carboxyl-terminus of HSP70-interacting protein). This E3 ligase -mediated AR ubiquitination has a much lower turnover efficiency, leading to the abnormal sustained accumulation of polyubiquitinated AR and decreased AR activity [60].

Considering the aforementioned information, CDK7-mediated AR S515 phosphorylation could provide a therapeutic opportunity to inhibit AR activity, to complement the second-generation antiandrogen therapy. It was reported that triptolide (TPL), a natural product from the medicinal plant *Tripterygium wilfordii Hook F.*, inhibits AR signaling pathways to suppress the proliferation of enzalutamide-resistant PCa cells [61]. TPL was found to inhibit the transactivation activity of AR and the CRPC-associated AR splice variants by inhibiting XPB/CDK7 and AR S515 phosphorylation. TPL significantly restrains the binding of AR to the promoter regions of its target genes, concurrently reducing the recruitment of TFIIH and RNA Pol II. Furthermore, at low doses, TPL diminishes the viability of PCa cells expressing AR or AR-Vs and exhibits a synergistic effect with enzalutamide in inhibiting CRPC cell survival in vitro [61].

### 4.2. Negative Regulation of AR Transcriptional Activity

In addition to their positive modulation of AR transcriptional activity, cyclins and CDKs also participate in the negative regulation of AR-dependent transcription. The serine 308 reside in the TAU1 region of the NTD was found to be phosphorylated by cyclin D3/CDK11p58 [62,63]. CDK11p58 is a short isoform of the Ser/Thr protein kinase CDK11 [64] that is activated by cyclin D3. This kinase-mediated S308 phosphorylation inhibits AR-dependent transcription and negatively regulates the androgen-dependent proliferation of PCa cells both in vitro and in vivo [63].

### 4.3. Positive Regulation of AR Nuclear Translocation

In addition to altering AR transcriptional activity, AR phosphorylation also regulates the subcellular localization of AR. It was found that EGF-induced downstream kinase activation results in ERK1/2-mediated phosphorylation at S515 in the NTD and PKC-mediated S578 phosphorylation in the DBD, resulting in an increase in AR transcriptional activity [65,66]. This is an example of growth-factor-induced ligand-independent activation of AR. Phosphorylation at these sites regulates the nuclear-cytoplasmic shuttling of AR and its interaction with the Ku-70/80 regulatory subunits of DNA-dependent protein kinase [65,67,68]. Eliminating AR S578 phosphorylation abolishes the EGF-activated AR transcriptional activity and increases nuclear retention of AR through its binding with Ku-70/80. Rapid release and rebinding of AR and other steroid receptors to DNA was found to be required for the steroid-receptor-activated transcription [69,70,71]. It was suggested that EGF-dependent S578 phosphorylation may have a similar function in regulating AR association and dissociation from DNA, through modulating the nucleus-cytoplasm shuttling. S578A mutation also causes hyperphosphorylated S515, suggesting that S578 phosphorylation limits S515 phosphorylation. S515A mutation, in contrast, can only slightly reduce EGF-induced AR activation but cannot completely eliminate it. It appears that the observed hyperphosphorylated S515 in the context of S578A mutation plays a minimal role in EGF-induced AR transactivation since S578A completely abolishes EGF-dependent induction. The results suggest that AR phosphorylation at S578 in the DBD-first zinc module regulates AR nuclear-cytoplasm shuttling and AR transactivation in response to EGF, ultimately promoting CRPC cell growth [65]. However, in triple-negative breast cancers (TNBC), AR S515 phosphorylation in combination with pERK1/2 was found to be associated with poor cancer-specific survival [72]. This phosphorylation was suggested to be a potential prognostic marker and therapeutic target for TNBC.

### 4.4. Negative Regulation of AR Nuclear Translocation

Intriguingly, p21-activated kinase 6 (PAK6) also regulates AR subcellular localization through phosphorylation at S578, but with a different outcome. Upon androgen stimulation, PAK6-mediated S578 phosphorylation obstructs AR translocation into the nucleus and enhances the association of AR with its E3 ligase MDM2, promoting ubiquitin-mediated degradation of AR and consequently inhibiting PCa growth. A decrease in PAK6 and AR co-localization in the cytoplasm and an increase in AR nuclear expression were observed in high-grade PCa, indicating a negative regulatory role of PAK6 in AR transactivation [73]. The two opposite outcomes from the same S578 phosphorylation are likely due to the formation of two different AR subcomplexes, depending on the kinases (PKC or PAK6) interacting with AR as well as other associated complex-specific factors.

Two other serine (S650 and S815) phosphorylation processes were identified to negatively regulate AR activity by disrupting AR nuclear localization. S650 is the sole residue known so far to undergo phosphorylation within the hinge region of AR [38,74,75,76,77,78,79,80]. Given its proximity to the DBD nuclear export signal, its involvement in nuclear-cytoplasmic shuttling was studied. It was reported that stress kinases MKK4/JNK and MKK6/p38 are directly involved in phosphorylating this site [81], and S650 phosphorylation exerts a negative influence on AR transcriptional activity by facilitating its nuclear export [81]. However, another study showed a different effect of S650 phosphorylation on AR activity regulation: it demonstrated that substituting S650 with alanine led to a 30% reduction in AR transcriptional activity, indicating that S650 plays a pivotal role in optimal AR transactivation [76].

Recently Yokobori et al. showed that PKCα-mediated AR phosphorylation at the S815 located in the LBD disrupts the DHT-induced nuclear translocation [82]. Expression levels of phosphorylated AR at S815 were observed to decrease following castration in the mouse prostate, indicating that the hormones secreted from the testes regulated AR phosphorylation at S815. The decrease in AR phosphorylation at S815 following castration was not recovered by testosterone injection, suggesting that S815 phosphorylation was mediated by a hormone secreted from the testes, but not testosterone. S815 phosphorylation levels progressively decrease as PCa worsens [82]. Mechanistically, phosphorylation at S815 suppresses oncogenic AKT activity in PCa cells following DHT activation. It is known that AR negatively regulates AKT activity and androgen deprivation activates AKT, which in turn inhibits antiandrogen efficacy in treating PCa [50,83,84]. However, the loss of S815 phosphorylation upon androgen deprivation is believed to partially contribute to AKT reactivation [82]. In addition to this, S815 phosphorylation was also reported to stimulate ER stress-induced cell death and modulate AR’s role in maintaining the prostate [85].

### 4.5. Positive Regulation of AR Protein Stability

Besides regulating AR transactivation and nuclear-to-cytoplasmic translocation, multiple proteins have been identified for their capacity to stabilize AR protein post-activation. PIM-1 is one such protein observed to have elevated expression levels in mouse models of prostate tumors and human PCa [86,87]. Two isoforms of PIM-1 kinase, namely PIM-1S and PIM-1L, modulate AR stability and transcriptional activity by distinctively phosphorylating AR at serine 215 (S215, formerly S213) and threonine 850 (T850), respectively. Both PIM-1S- and PIM-1L-mediated AR phosphorylation promote the recruitment of E3 ubiquitin ligases. While S215 phosphorylation (by PIM-1S) promotes AR degradation, T850 phosphorylation appears to stabilize the protein. It was found that two different E3 ubiquitin ligases were recruited by the two different phosphorylation sites, leading to different outcomes [88]. Specifically, only PIM-1L has the potential to phosphorylate T850 in the LBD of AR. T850 phosphorylation recruits the non-proteolytic E3 ubiquitin ligase RNF6, which does not destabilize AR. The RNF6-induced ubiquitin chains in AR are non-canonical K6- and K27-linked, and atypically ubiquitinate AR primarily on the K845 amino acid [89]. Unlike K11- and K48-linked ubiquitination, which mainly results in the degradation of the substrates by 26S proteasome, K6 and K27 linkage have been associated with protein recruitment where the ubiquitin chain serves as an interaction platform to allow protein binding [90]. The RNF6-induced non-canonical polyubiquitination of AR recruits its coactivators to a subset of AREs promoting AR transcriptional activity under conditions of low androgen [88,89,91]. The phosphor-mimicking T850D mutation was found to protect AR from PIM-1S-promoted AR degradation, thereby stabilizing the protein [88].

In addition to T850 phosphorylation, the stabilization of AR protein is also facilitated by CDK5 and its coactivator-p35-mediated S81 phosphorylation. The CDK5-dependent AR stabilization causes accumulation of AR in the nucleus and protects it from ubiquitin-proteasome degradation [42]. CDK5 also phosphorylates the transcription factor signal transducer and activator of transcription 3 (STAT3) at residue S727 and promotes the interaction between phosphorylated STAT3 and AR. Through controlling the phosphorylation-dependent STAT3-AR interaction, CDK5 also indirectly stabilizes the AR protein and activates its transcriptional activity. Correspondingly, clinical evidence reveals that the level of p-S727-STAT3 is significantly correlated with the levels of upstream regulators (CDK5 and p35) and downstream protein (AR) [92].

### 4.6. Negative Regulation of AR Protein Stability

Serine phosphorylation at the NTD S215 (formerly S210/213) was found to negatively regulate AR protein stability. S215 phosphorylation of AR is mediated by AKT [93]. A flavonolignan compound isosilybin B, a traditional botanic agent from milk thistle seed known to inhibit PCa cell proliferation and PSA secretion [94], was found to increase AKT phosphorylation, resulting in an increased AR phosphorylation at S215 [95]. Increased S215 phosphorylation suppresses AR transcriptional activity, prevents androgen-induced AR nuclear localization, and inhibits cell growth in LNCaP, 22Rv1, and LAPC4 cell lines [95,96,97]. Mechanistically, isosilybin B treatment was found to facilitate the formation of a complex between AR, AKT, and MDM2, to promote phosphorylation-dependent AR ubiquitination and its degradation by proteasome. The half-life of AR protein was reduced by half when treated with isosilybin B, suggesting that S215 phosphorylation is important for promoting protein turnover [95]. As mentioned above, Linn et al. also demonstrated that PIM-1S induced S215 phosphorylation destabilizes AR by recruiting the ubiquitin E3 ligase MDM2 and promoting AR degradation in a cell-cycle-dependent manner [98]. The unphosphorylated S215 mutant is significantly more stable and is resistant to PIM-1S-induced destabilization [98,99,100]. Notably, S215 phosphorylation is cell-cycle dependent and is observed to increase during the G2 and M phase, aligning with elevated PIM-1S expression. Mutations of S215 abolished PIM-1S-promoted AR degradation during the M phase, suggesting that PIM-1S plays a role in modulating AR degradation during mitosis [98].

### 4.7. Phosphorylation Sites with Multiple Effects

Apart from regulating a single functional aspect of AR activity, several phosphorylation sites have the capacity to regulate multiple AR functions. Y267 and Y363 are two such closely located tyrosine residues in the NTD of AR, known to become phosphorylated by Cdc42-associated tyrosine kinase Ack1 in an androgen-independent manner. Activated-Ack1-mediated Y267 and Y363 phosphorylation promotes AR nuclear translocation, its recruitment to ARE, androgen-regulated gene expression, and the androgen-independent growth of LNCaP and LAPC4 xenografts [101,102]. Expression of the constitutively active mutant of Ack1 (caAck1) in LNCaP cells increased AR binding to the KLK3 (PSA) gene promoter and led to the upregulation of PSA expression in the androgen-depleted conditions [103].

S215 and S792 are two other such sites located in the NTD and LBD of AR, respectively. S215 (as mentioned above) and S792 phosphorylation are dependent on PI3K/AKT signaling [93,104]. Activation of PI3K-Akt signaling via insulin-like growth factor-1 (IGF-1) leads to the activation of downstream kinases p70S6K or AKT [105]. AKT represses AR function by phosphorylating AR at the consensus sites S215 and S792. Phosphorylation at either S215 or S792 or both prevents ligand binding, inhibits androgen-induced nuclear translocation, decreases AR transcriptional activity, and promotes AR protein degradation via the ubiquitin–proteasome system [104].

Lastly, Y534 is another multifunctional phosphorylation site present in the NTD of AR that is phosphorylated by Src family kinases [40,102]. Src family kinases and Ack1 phosphorylate AR (Y534 and Y267, respectively) promote CRPC growth [40,102,106]. These kinases are overexpressed in CRPC, and their levels correlate with poor prognosis [41,106]. It was reported that EGF stimulation induces AR Y534 phosphorylation and increases AR transcriptional activity [40]. Y534 phosphorylation also promotes nuclear localization of AR and its recruitment to the KLK3 gene promoter [40]. In the absence of androgen, Src activation can trigger androgen-independent AR-dependent gene expressions. In addition, Src also enhances AR binding to non-canonical AR binding sites that do not have the known ARE sequences. These non-canonical AR binding sites share binding consensus sites for FOXO1, topoisomerase IIβ (TOP2B), and ZNF217, suggesting that Src-activated AR might either compete for their binding sites or form complexes with these factors. Indeed, the differential expression of these three factors has been observed to facilitate PCa progression. The downregulation of FOXO1 and the upregulation of TOP2B and ZNF217 correlate with earlier metastatic recurrence and are associated with poor survival in CRPC patients. Increased AR Y534 phosphorylation thus sensitizes AR to low intracrine androgen levels, to facilitate CRPC progression [107].

### 4.8. Phosphorylation Sites with Unknown Functions

In addition to the above-mentioned phosphorylation sites, mass spectrometry identified several other tyrosine residues that could function as potential AR phosphorylation sites, including Y307, Y46, Y357, Y362, Y393, Y551, and Y915 [40]. These tyrosine residues were primarily mapped to exon 1 of AR, which is conserved between full-length AR and variants, suggesting that they might play a role in the regulation of both forms of AR [108]. However, the detailed function and mechanism of these phosphorylation sites is undefined and warrants further investigation.

### 4.9. Dephosphorylation

Androgen binding can enhance the phosphorylation state of AR either by negatively regulating the ability of LBD to bind phosphatases or by inducing an AR conformation that is resistant to phosphatase action [109]. Phosphorylation of AR is a reversible process and currently only two protein phosphatases have been described to interact with and dephosphorylate AR. Protein phosphatase 2A (PP2A) is a serine/threonine phosphatase and a potent tumor suppressor known to dephosphorylate AR [109,110]. PP2A regulatory and catalytic subunits were found to be present in an androgen-induced AR complex, through mass spectrometry [109]. PP2A binds to the C-terminal half of AR and dephosphorylates several serine residues at the NTD, including S81 [109]. PP2A-mediated dephosphorylation inhibits AR transcriptional activity. Decreased PP2A activity is associated with the enhanced androgen-independent growth ability of PCa cells [111]. Downregulation of PP2A has also been shown to enhance phosphorylation of AKT, ERK, and BAD (BCL2-associated agonist of cell death); increase expression of cyclins (A1/D1); and decrease expression of cyclin inhibitor (p27), ultimately potentiating proliferation and survival signaling in cells grown in androgen-deprived conditions [111].

Protein phosphatase 1 (PP1) has demonstrated an opposite effect on AR activity compared to PP2A. PP1 dephosphorylates S650 in the hinge region of AR, consequently increasing its AR expression levels, nuclear localization, and transcriptional activity [112]. It was reported that protein phosphatase 1 catalytic subunit (PP1α) contributes significantly to elevated AR expression (primarily by binding to AR LBD), thereby reducing ubiquitination and subsequent degradation of the receptor. Inhibition of PP1α by tautomycin has been observed to elevate the phosphorylation of AR ubiquitin ligases SKP2 and MDM2 at sites that enhance their functionality, proposing a plausible mechanism through which PP1α may suppress AR degradation [113]. Moreover, PP1 inhibition causes a significant decrease in the nuclear localization of AR, by dramatically increasing phosphorylation at S650 [112]. Collectively, these findings suggest that PP1α potentially plays a role in maintaining AR protein stability and nuclear localization following androgen deprivation therapies. Targeting either PP1α itself or disrupting the interaction between AR and PP1α could prove efficacious in managing CRPC [113].

## 5. Ubiquitination

Ubiquitin is a conserved, 76-amino-acid protein often found covalently conjugated to proteins to regulate their activity [114]. While AR ubiquitination can result in advancement to this classical pathway, recent investigations have focused on the role of AR non-classical ubiquitination in promoting CRPC. Here, we discuss how classical and/or non-classical ubiquitination of AR influence PCa progression.

### 5.1. Negative Regulation of AR Function via Proteolytic Ubiquitination

Studies identifying proteolytic E3 ubiquitin ligases and inducers promoting AR degradation have focused on observing in vitro and in vivo reductions in AR expression and/or transcriptional activity. This ubiquitin-mediated degradation of AR ultimately causes PCa growth and proliferation attenuation. Currently identified AR proteolytic E3 ubiquitin ligases include MDM2 [115], DDB2-CUL4A complex [116], PMEPA1-bound NEDD4 [117], and SKP2 [118]. Additionally, the nuclear export signal of AR (NESAR) located at the LBD can act as a signal for AR ubiquitination and proteasomal degradation in PC3 cells [119]. SPOP, a frequently mutated E3 ubiquitin ligase in PCa, can bind to and ubiquitinate AR in prostate adenocarcinoma cells [120], while prostate adenocarcinoma-associated SPOP mutations abolish this activity. SPOP-mediated degradation of AR requires a ^645^ASSTT^649^ motif in the hinge domain of AR. This degradation does not affect prostate cancer-derived hinge-domain-deficient AR splicing variants. Androgen binding to AR attenuates this degradation by diminishing AR-SPOP interaction [120]. STUB1 (CHIP, C-terminus of HSP70 interacting protein), a co-chaperone protein, and E3 ubiquitin ligase form a complex with HSP70 which is a chaperone-assisting folding and maturation of AR [121]. CHIP dissociates AR/AR-V7 from HSP70, leading to AR/AR-V7 protein ubiquitination and degradation [60,122,123,124].

After identifying these E3 ubiquitin ligases, follow-up studies on these ligases focused on identifying proteins that can induce these ligases to ubiquitinate AR. Many studies identifying these inducers focused on MDM2 inducers. As mentioned previously, AKT-mediated AR phosphorylation at Ser 215 and Ser 792 increases the interaction between MDM2 and AR, promoting AR ubiquitination and degradation and leading to suppression of AR activity [115]. Kinase PIM-1S, as mentioned in the Phosphorylation Section, also phosphorylates AR S215 to promote MDM2 recruitment [98]. Additionally, beta arrestin 2 acts as an AR corepressor and serves as a scaffold to recruit MDM2 for AR degradation [125].

### 5.2. Deubiquitination and Inhibition of Ubiquitination

Considering the previous discussion, a viable therapeutic strategy for treating PCa is to induce AR proteolytic ubiquitination, resulting in decreased AR transcriptional activity and ultimately halting PCa cell growth. Regarding this strategy, there are many studies focusing on elucidating proteins that can reverse this ubiquitination, to preserve AR transcriptional activity in PCa. Ubiquitin-specific proteases (USPs) 7, 12, 14, and 26 preserve AR by deubiquitinating AR and preventing degradation [126,127,128,129]. Meanwhile, USP7 directly deubiquitinates AR upon AR-androgen binding [128]. USP12 deubiquitinates AKT phosphatase PHLPP. PHLPPL reduces phospho-AKT levels, which in turn downregulates AR Ser215 phosphorylation, preventing MDM2 recruitment and subsequent AR degradation [130]. Interestingly, pVHL (von Hippel–Lindau, a subunit of a multiprotein E3 ubiquitin ligase complex known to target HIF1α for proteasomal degradation) inhibits AR activity through deubiquitinating AR instead of ubiquitinating AR. This de-ubiquitination does not affect AR turnover [131].

As a corollary to identifying proteins that de-conjugate ubiquitin from AR, some studies focused on identifying proteins that directly inhibit E3 ubiquitin ligase function or bind to AR to prevent its ubiquitination. PrLz (prostate leucine zipper gene) directly binds to AR and stabilizes AR through competing with MDM2 for AR binding, thereby reducing AR ubiquitination and degradation and enhancing AR activity [132]. Similarly, BMI1 (a polycomb group protein) also directly interacts with AR and stabilizes the AR protein via competitive inhibition of MDM2-mediated ubiquitination [133]. HOTAIR is androgen-repressed lncRNA. Under castration conditions, HOTAIR levels increase. This binds AR and blocks AR-MDM2 interaction, thereby stabilizing AR protein. It was found that HOTAIR expression is sufficient to drive androgen-independent AR activation under castration conditions [134]. Lyn is an Src family kinase. Unlike the tyrosine kinases Ack1 and Src mentioned above (that directly phosphorylate AR tyrosine residues and enhance AR activity), Lyn maintains AR protein stability. Under castration conditions, Lyn is required for maintaining the interaction between AR and the molecular chaperone HSP90, and it prevents proteasomal degradation of ligand-unbound AR [135]. Another heat-shock protein HSP27 also plays a role in stabilizing AR protein. Upon androgen stimulation, HSP27 displaces HSP90 from a complex with AR and chaperones AR into the nucleus to activate transcription. Knockdown of HSP27 was found to disrupt AR-HSP90 association and increase AR-MDM2 association, inducing AR ubiquitination and degradation [136]. Additionally, parathyroid hormone-related protein (PTHrP) can reduce AR ubiquitination by leveraging EGFR and Src pathways to phosphorylate AR Tyr534 and reducing AR interaction with the E3 ubiquitin ligase CHIP. This results in the accumulation of AR protein and enhanced PCa growth at low levels of androgen [137]. Splicing factor PRP8 was found to be an AR cofactor that interacts with the nuclear export signal NESAR within the LBD of AR and reduces AR ubiquitination [138]. This study provides a link between AR signaling and splicing machinery.

#### 5.2.1. Proteolytic Ubiquitination and Positive Regulation of AR Transcription

Proteolytic ubiquitination is usually associated with protein degradation and the inhibition of protein activity, as mentioned above. However, positive effects of proteosome-mediated protein degradation on transcriptional activities have also been reported for many transcription factors and coactivators [139,140,141]. It is believed that proteolytic ubiquitination can function to control transcription factor chromatin binding, residency time, and coactivator exchange on promoter DNA, to promote transcription [139]. Zipper-interacting-protein-kinase (ZIPK)-regulated MDM2-mediated AR ubiquitination is one such example. ZIPK functions as an AR binding partner and a coactivator. It forms a single complex at the chromatin with AR and MDM2. ZIPK phosphorylates MDM2 in vitro and cooperates with MDM2 to promote AR proteolytic ubiquitination. This degradation is necessary for the AR cyclic transcription complex assembly. Knockdown of ZIPK results in accumulation of AR at the PSA enhancer and promoter regions, which appears to be non-functional [142]. In this study, MDM2 appears to activate AR transcriptional activity through promoting chromatin-bound AR turnover [142], which is different from other studies showing a repressive effect of MDM2.

Siah2 is another E3 ubiquitin ligase that targets AR degradation while promoting its transcriptional activity [143]. Siah2 was found to ubiquitinate a select pool of chromatin-bound and corepressor-NCOR1-bound transcriptionally inactive AR for degradation. This activity promotes the expression of select AR target genes involved in lipid metabolism, cell motility, and proliferation, and it is required for PCa cell growth under androgen-deprived conditions [143]. A RNA helicase DHX15 was found to regulate the ability of Siah2 to ubiquitinate AR by complexing with AR and Siah2 and enhancing Siah2 activity [144]. DHX15 is overexpressed in human PCa and correlates to PSA recurrence. Knocking down DHX15 reduces PCa cell growth and proliferation in vitro and in vivo.

#### 5.2.2. Non-Proteolytic Ubiquitination

More recent investigations into AR ubiquitination have highlighted an important role of the non-classical (non-proteolytic) ubiquitination of AR in regulating PCa progression. Interestingly, many of these investigations imply that non-classical ubiquitination usually promotes AR transcriptional activity. Our group has recently shown that E3 ubiquitin ligase TRAF4 (TNF receptor-associated factor 4) is overexpressed in metastatic CRPCs. It ubiquitinates AR K913 (identified in vitro and in cells) via ubiquitin’s K27 linkage. This ubiquitination increases AR-FOXA1 interaction and upregulates a distinct AR transcriptional program that is activated by AR in CRPC cells but not in androgen-sensitive cells, thereby promoting CRPC cell growth [90]. Another E3 ubiquitin ligase RNF6 is also overexpressed in hormone-deprived PCa tissue; it mediates AR ubiquitination in K845 residue (both in vitro and in cells) and facilitates AR binding to cofactors at a subset of AREs [91]. As described above in the Phosphorylation Section, RNF6 is recruited to AR when kinase PIM-1L phosphorylates AR T850 to promote AR activity under low-androgen conditions [98].

Another two E3 ubiquitin ligases were also found to promote AR transcriptional activity, but the underlying mechanisms remain elusive. TRIM68 promotes AR transcriptional activity upon androgen treatment. This upregulation may be partially due to the interaction between TRIM68 and AR coactivators TIP60 and p300. It was speculated that TRIM68 may ubiquitinate the AR corepressor to promote corepressor–coactivator switching upon androgen stimulation [145]. Finally, E3 ubiquitin ligase enhanced at puberty 1 (EAP1) was also found to enhance AR transcriptional activity. However, EAP1 does not directly bind AR. Further investigations into how EAP1 accomplishes this will need to be conducted to understand the mechanism [146].

### 5.3. SUMOylation

Small ubiquitin-like modifiers (SUMO), also known as sentrin and SM3tp, are a family of proteins often found covalently bonded to other proteins as a post-translational modification [147]. Mammals express three major SUMO isoforms: SUMO1, SUMO2, and SUMO3. SUMO2 and SUMO3 are highly similar (sharing about 97% identical residues) and cannot currently be distinguished by Western blots [148]. The covalent conjugation of SUMO1 and SUMO2/3 to AR leads to broad, target-specific changes in AR’s transcriptional activity [149,150,151,152]. SUMO1 conjugation appears to have a different function compared to SUMO2/3 [153]. SUMO specific proteases (SENPs) reverse this covalent conjugation [154]. Here, we review different aspects of AR SUMOylation and how this modification affects PCa progression.

The SUMO E2 conjugating enzyme Ubc9 was found to interact with AR and promote SUMO1 conjugation in K386 and K520 residues (identified in cell-based assays) at the NTD of AR, with K386 being the major SUMOylated site. This SUMOylation represses AR transcriptional activity [155]. Further genome-wide gene expression and ChIP-seq analyses using the SUMOylation-defective AR K386/502R mutant indicate that SUMOylation does not simply repress AR activities on all target genes. Instead, AR SUMOylation selectively regulates cell proliferation and apoptosis pathway genes [150]. Interestingly, the two SUMO conjugation sites K386 and K520 are present in synergy control (SC) motifs, which are four amino acids often preceded and/or followed by nearby proline or glycine residues [156]. The SC motifs were found present in multiple transcription factors and inhibit transcription factor activities when bound to multiple cognate response elements [156,157]. Mutations of P390 and G524 within the SC motifs nearby the SUMOylation sites were frequently found in androgen-insensitive syndrome and CPRC, respectively [156]. It was reported that mutation of these adjacent sites leads to a partial loss of AR SUMOylation [156].

A study on Daxx revealed one of the underlying mechanisms for SUMOylation-mediated AR transcription repression [158]. Daxx was originally identified as a Fas-cell-surface-receptor-binding protein that enhances Fas-mediated apoptosis and activates the Jun N-terminal kinase (JNK) pathway [159]. It was found that Daxx was recruited by SUMOylated AR (SUMOylation at K386/K520 residues) and repressed AR DNA binding activity through interaction with the AR DBD, thereby inhibiting AR transcriptional activity [158].

Considering the repression effect imposed by SUMOylation, a number of studies have focused on proteins that deconjugate SUMO1 from AR or inhibit SUMO1 binding to AR, which ultimately enhances AR transcriptional activity. After androgen stimulation and subsequent AR translocation into the nucleus, AR recruits SUMO-specific protease 1 (SENP1) to rapidly remove SUMO1 groups from AR in the nucleus. Interestingly, SENP1 activates AR transcriptional activity on multiple AREs but not single ARE-containing promoters [154]. AR also drives SENP1 transcription [160]. siRNA against SENP1 disrupted this positive feedback loop and significantly reduced androgen-driven LNCaP PCa cell growth [160]. In addition to deSUMOylating AR, SENP1 also targets other AR-associated transcription factors and coregulators. SENP1 reverses the pioneer transcription factor forkhead box A1 (FOXA1) SUMOylation, which allows FOXA1 to interact and facilitate AR binding to chromatin [161]. It also deSUMO1ylates HDAC1 (histone deacetylase 1) (a known AR transcriptional activity repressor) but not the coactivators SRC-1 and p300 [162]. DeSUMOylation inhibits HDAC1 repressive activity and therefore enhances AR-dependent transcription [162]. Additionally, SENP1 was found to play a role in cadmium (a potential prostate carcinogen and an androgen mimic)-induced AR transcriptional activity [163]. Cadmium exposure upregulates SENP1 expression and decreases AR SUMOylation, further promoting PCa cell proliferation [163].

Based on the role of AR SUMO1ylation in regulating AR activity, inhibition of the ability of SENP1 to deSUMOylate AR would be a potential therapeutic strategy for inhibiting PCa progression. Huang et al. demonstrated that triptolide, the natural product mentioned above inhibiting AR S515 phosphorylation, also inhibited LNCaP and PC3 cell proliferation through downregulating SENP1 expression. In addition, triptolide also suppresses the expression of AR and c-Jun, a major component of transcription factor AP-1, which also coactivates AR [164]. Consequently, triptolide significantly inhibits PCa cell proliferation and xenograft tumor growth and induces cell apoptosis [164].

Several studies have identified additional proteins other than the well-known E3 SUMO ligase PIAS1 that promote AR SUMOylation. Sharma et al showed that hZimp10 (human zinc finger-containing, Miz1, PIAS-like protein on chromosome 10) overexpression modestly promotes AR SUMOylation at the K386/520 sites. Interestingly, this study indicates that hZimp10 is an AR coactivator. Mutation of AR SUMOylation sites abolished hZimp10 coactivation activity. However, it is not clear how hZimp10-enhanced AR SUMOylation increases AR activity, in contrast to other reports. Notably, hZimp10 co-localizes with AR SUMO-1 at the DNA replication foci during the cell-cycle S-phase, suggesting a potential role of hZimp10 in both chromatin assembly and the maintenance of chromatin [165]. HDAC4 has been shown to inhibit AR transcriptional activity by binding to AR and promoting AR SUMOylation in vivo and in vitro. This study revealed a deacetylase-independent function of HDAC in PCa cells [166].

Unlike SUMO1, which usually modifies a substrate as a monomer, SUMO2/3 can form a poly-SUMO chain [167,168]. The PIAS1-mediated SUMO1 modification of AR reduces AR transcriptional activity without affecting its subcellular localization and DNA-binding [169,170,171]. It was reported that SUMO2/3 modification is also involved in regulating AR function [172]. PIAS1 itself is modified by SUMO3, and SUMOylated PIAS1 interacts with AR SUMO target site K386 and the ubiquitination target site K845 to form a binary complex. This interaction distributes AR to the cytoplasm and recruits MDM2 to promote AR ubiquitination and degradation [172]. PIAS1 was also found to possess SUMO E3 ligase-independent functions, allowing it to act as a chromatin-bound-AR coregulator and regulate (either to activate or repress) androgen-regulated genes in a target-gene-selective fashion [171,173,174]. PIAS1 was found to interact with AR, FOXA1, and SUMO2/3 on chromatin. Androgen exposure increases the co-occurrence of SUMO2/3 at AR-, PIAS1-, and FOXA1-shared genomic locations, suggesting that the regulatory effects of PIAS1 at these genomic loci may receive a partial contribution from the enhanced SUMO2/3 conjugation of AR and/or associated proteins [174]. It was reported that androgen (testosterone) but not anti-androgen bicalutamide induces AR SUMO2/3 modification. Furthermore, heat stress also induces rapid and reversible AR-SUMO2/3 modifications. This causes AR detachment from chromatin and subsequent accumulation of SUMO2/3-conjuated AR in the nuclear matrix compartment. The mutation of AR K386 and K520 SUMOylation sites impairs this solubility. Allowing cells to recover after heat stress causes AR-SUMO2/3 to re-occupy AR target genes, suggesting a role of SUMO2/3 in mediating stress-induced AR sequestration to the nuclear matrix, which is reversible upon stress removal [172]. Interestingly, SUMO-3 inhibits exogenous AR transcriptional activity in AR-negative PC3 cells. This SUMO-3 inhibition activity does not require the two functional AR SUMOylation sites [172].

Similar to SENP1, PIAS1 expression is also upregulated by androgen [175]. PIAS1 expression is positively correlated with AR expression in PCa cell lines and PCa patient tissues. High PIAS1 levels predict shorter relapse-free survival [175]. PIAS1 depletion in combination with abiraterone or enzalutamide treatment was found to be more effective at decreasing cell proliferations than single drug treatment, indicating that PIAS1 might be a promising candidate for combination therapies [175].

## 6. Conclusions

AR PTMs heavily influence AR transcriptional activity and subsequent PCa progression. Moreover, some of these PTMs activate AR functions despite androgen deprivation. As discussed previously, acetylation generally improves AR transcriptional activity by improving AR binding to DHT and other TFs, while methylation could attenuate or enhance AR transcriptional activity depending on the specific methyltransferases recruited. Furthermore, ubiquitination can either prevent AR transcriptional activity (by causing MDM2-mediated degradation) or activate AR transcriptional activity through non-classical modification mechanisms, regardless of androgen deprivation. Additionally, SUMO1 generally hinders AR transcriptional activity, while deSUMOylation mediated by SENP1 activates AR activity. Phosphorylation is more complicated, and it can stimulate or attenuate AR transcriptional activity in a phosphorylation-binding-site-dependent manner.

As discussed in this review, many AR PTM modifiers are aberrantly regulated and could contribute to the development and progression of PCa. Many of these studies clearly identify unique AR PTM regulators as promoters of castration resistance. Controlling AR PTMs during ADT to modulate AR activity would represent one therapeutic strategy for combating PCa. This review highlights several areas where further investigation should be heavily encouraged. For example, further investigations should be conducted to identify regulators of AR methylation and consider how these regulators impact PCa progression. Furthermore, several regulators of AR PTMs, such as SENP1 and TRAF4, have clearly been identified as viable inhibitory therapeutic targets for PCa. Some of the modifiers, such as MDM2, appear to have opposite outcomes depending on the kinases and subcomplexes AR associate with. It may be risky to simply target or activate MDM2. The upstream kinases associated with specific AR regulation or combined with downstream modifiers may represent better therapeutic targets.

Elucidating the feasibility of therapy that combines inhibiting these regulators with current PCa treatments should be the next objective for follow-up studies. Furthermore, combination therapy development should focus on inhibiting multiple mechanisms promoting castration resistance for full-length AR and AR splicing variants. This review highlighted numerous oncogenes enabling AR’s evasion from ADT. However, it is unclear whether some of these PTM modifiers become alternatively activated to promote CRPC when the therapeutic inhibition of certain modifiers is applied. Identifying these potential compensatory oncogenes should also aid combination therapy development.

## Data Availability

Not applicable.
